# Thyrotropin-Releasing Hormone (TRH) and Somatostatin (SST), but not Growth Hormone-Releasing Hormone (GHRH) nor Ghrelin (GHRL), Regulate Expression and Release of Immune Growth Hormone (GH) from Chicken Bursal B-Lymphocyte Cultures

**DOI:** 10.3390/ijms21041436

**Published:** 2020-02-20

**Authors:** Santiago Pech-Pool, Laura C. Berumen, Carlos G. Martínez-Moreno, Guadalupe García-Alcocer, Martha Carranza, Maricela Luna, Carlos Arámburo

**Affiliations:** 1 Departamento de Neurobiología Celular y Molecular, Instituto de Neurobiología, Campus Juriquilla, Universidad Nacional Autónoma de México, Querétaro 76230, Mexico; agronomiapech@hotmail.com (S.P.-P.); macasa@unam.mx (M.C.); 2 Posgrado en Ciencias Químico-Biológicas, Facultad de Química, Universidad Autónoma de Querétaro, Centro Universitario, Querétaro 76010, Mexico; lcbsq@yahoo.com (L.C.B.); guadalugar@yahoo.com.mx (G.G.-A.); 3 Instituto de Neurobiología, Universidad Nacional Autónoma de México, Boulevard Juriquilla 3001, Querétaro 76230, Mexico

**Keywords:** bursa of fabricius, growth hormone, bursal B-lymphocytes, TRH, somatostatin, GHRH

## Abstract

It is known that growth hormone (GH) is expressed in immune cells, where it exerts immunomodulatory effects. However, the mechanisms of expression and release of GH in the immune system remain unclear. We analyzed the effect of growth hormone-releasing hormone (GHRH), thyrotropin-releasing hormone (TRH), ghrelin (GHRL), and somatostatin (SST) upon GH mRNA expression, intracellular and released GH, Ser133-phosphorylation of CREB (pCREB^S133^), intracellular Ca^2+^ levels, as well as B-cell activating factor (BAFF) mRNA expression in bursal B-lymphocytes (BBLs) cell cultures since several GH secretagogues, as well as their corresponding receptors (-R), are expressed in B-lymphocytes of several species. The expression of TRH/TRH-R, ghrelin/GHS-R1a, and SST/SST-Rs (Subtypes 1 to 5) was observed in BBLs by RT-PCR and immunocytochemistry (ICC), whereas GHRH/GHRH-R were absent in these cells. We found that TRH treatment significantly increased local GH mRNA expression and CREB phosphorylation. Conversely, SST decreased GH mRNA expression. Additionally, when added together, SST prevented TRH-induced GH mRNA expression, but no changes were observed in pCREB^S133^ levels. Furthermore, TRH stimulated GH release to the culture media, while SST increased the intracellular content of this hormone. Interestingly, SST inhibited TRH-induced GH release in a dose-dependent manner. The coaddition of TRH and SST decreased the intracellular content of GH. After 10 min. of incubation with either TRH or SST, the intracellular calcium levels significantly decreased, but they were increased at 60 min. However, the combined treatment with both peptides maintained the Ca^2+^ levels reduced up to 60-min. of incubation. On the other hand, BAFF cytokine mRNA expression was significantly increased by TRH administration. Altogether, our results suggest that TRH and SST are implicated in the regulation of GH expression and release in BBL cultures, which also involve changes in pCREB^S133^ and intracellular Ca^2+^ concentration. It is likely that TRH, SST, and GH exert autocrine/paracrine immunomodulatory actions and participate in the maturation of chicken BBLs.

## 1. Introduction

The expression and release of growth hormone (GH) from the pituitary somatotrophs are regulated by several hypothalamic neuropeptides, such as growth hormone releasing hormone (GHRH), ghrelin (GHRL), thyrotropin-releasing hormone (TRH), and somatostatin (SST) [1,2,3]. After binding with their respective receptors in the somatotrophs, these hormones promote or inhibit the activation of other intracellular messengers and transcription factors, such as Ca^2+^ levels and the cAMP response element-binding (CREB), which are required for regulating GH expression and release [4]. The endocrine functions of GH include tissue growth, metabolic regulation, and homeostasis; its actions can be exerted directly, by GH receptor (GH-R) activation, or indirectly, through its classical mediator insulin-like growth factor 1 (IGF-1) [5]. In addition to the pituitary gland, it is now accepted that GH and GH-R are ubiquitously expressed in a diversity of tissues within the nervous, reproductive, and immune systems, among others, suggesting that GH has autocrine and/or paracrine effects in these extrapituitary expression sites [5,6].

In the immune system, GH stimulates the growth of primary and secondary lymphoid organs [7,8], where it induces lymphocyte proliferation and the production of cytokines and other immune factors [9,10]. In addition, GH also acts as a cytokine [11] and stimulates the innate and adaptative immune responses [12]. Moreover, GH is expressed in immune cells of various species, such as canine lymph nodes [13], human peripheral blood lymphocytes [14], rat, mouse, and bovine splenocytes [15,16], as well as in several immune cell lines (H-9 T cells and IM-9 B cells) [17].

In the chicken immune system, the expression and presence of GH mRNA and protein have both been detected in the spleen, thymus, and in bursa of Fabricius (BF) [18,19]. The BF is a primary lymphoid organ, exclusive of avian species, where bursal B-lymphocytes (BBLs) mature and develop, and after maturation they migrate to secondary lymphoid organs [20]. It has been described that GH mRNA is mainly expressed in lymphocyte progenitor cells in the bursal cortex, whereas the GH protein is more abundant in BBLs located in the medulla of BF [21], suggesting that GH could participate in BF development and function [18]. Furthermore, it was shown that, during embryogenesis, bursal GH-IR colocalized considerably with IgM-expressing cells but scarcely with IgG-producing lymphocytes, whereas the opposite was found after hatching, indicating that autocrine/paracrine actions of GH might be involved during the differentiation and proliferation of BBLs in BF [22]. It is also documented that GH content changes during the different stages of this organ and is related with the apoptotic waves that occur during BF development and involution, implying stage specific functions during ontogeny [22,23]. In addition, the administration of GH to BBL cultures (in vitro) increased cell viability, and decreased both caspase-3 activity and number of apoptotic cells, through the involvement of PI3K/Akt and Bcl-2 signaling pathways [24].

The expression of GH in immune cells suggests its participation as immunomodulator. However, the mechanisms that regulate the expression and release of GH in the immune system are poorly understood. It has also been reported that TRH and SST, as well as their receptors (TRH-R and SST-Rs), are expressed in the immune cells of various species [25,26]. TRH and SST can both influence the expression of several immune factors, thus indicating that they may play a role as immunomodulatory peptides [26,27]. In this work, we analyzed the participation of TRH and SST in the regulation of bursal GH expression and release in cultured BBLs from chicken BF, as well as their effect in the activation of CREB, the modulation of intracellular Ca^2+^ levels, and the expression of B-cell activating factor (BAFF) cytokine, which are important in several processes that are related to B-cell proliferation and survival [28]. Our results indicate that TRH and SST, and their receptors, are locally expressed in BBLs, and are involved in the regulation of immune GH synthesis and release in these bursal cells, implying paracrine and/or autocrine mechanisms participating in its immunomodulatory functions. Understanding the basic processes that are involved in GH regulation in avian B-lymphocytes and its involvement in the modulation of the immune response could eventually have some applications in the poultry breeding industry.

## 2. Results

### 2.1. Characterization and Viability of BBLs Cultures

**Figure 1:**
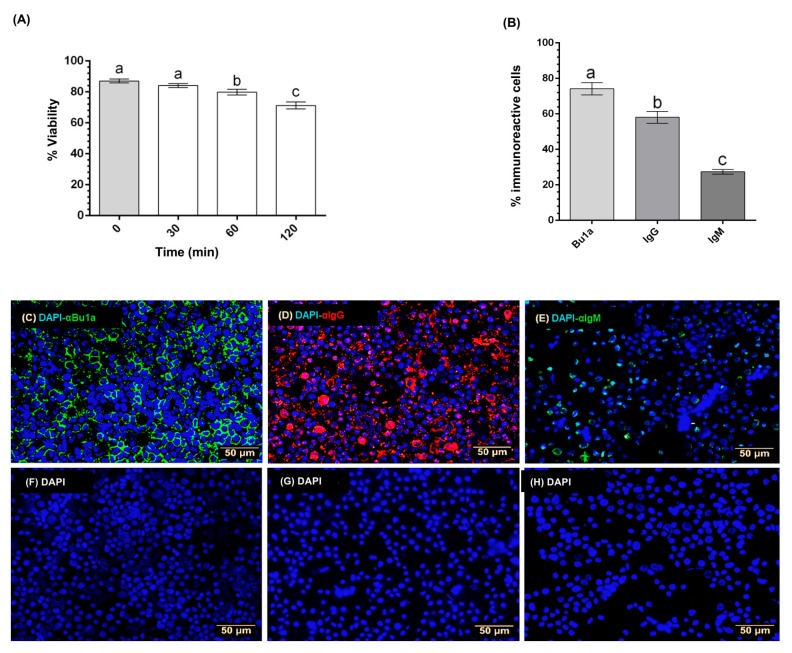
Characterization of bursal B-lymphocyte (BBL) cultures. Cell viability of BBLs was determined by the trypan blue exclusion method over 120 min. (**A**). Each bar represents mean ± SEM, *n* = 3. Groups with different letters are significantly different (*p* < 0.0001) by using one-way ANOVA and Dunnett´s *post-hoc* test. Cell subpopulations were characterized by ICC using primary antibodies to detect whole B cells (α-Bu1a) (**C**), mature B cells (α-IgG) (**D**), and immature B-cells (α-IgM) (**E**). DAPI staining was used to detect cell nuclei. Negative controls were prepared in the absence of primary antibodies (**F**–**H**). The proportion of BBLs subpopulations was calculated dividing the number of immunoreactive cells to each antibody between total number of DAPI reactive cells (**B**). Each bar represents mean ± SEM, *n* = 3. Groups with different letters are significantly different (*p* < 0.001) by using one-way ANOVA and Dunnett´s *post-hoc* test.

The cell viability of BBL cultures incubated in RPMI 1640 media alone was determined by the trypan blue exclusion method, along several incubation times (0–120 min.). BBLs viability decreased in a time-dependent fashion, as shown in Figure 1A: 87.01 ± 1.25% at time 0, 84.12 ± 1.21% after 30 min., 79.76 ± 1.82% after 60 min., and 71.21 ± 2.21% after 120 min. Specific markers for total B-cells (Bu-1a), as well as mature (IgG) and immature (IgM) B-lymphocytes were determined by immunocytochemistry (ICC) to characterize the proportion of BBLs subpopulations in the cultures. Figure 1B shows that, at 60 min. of incubation, 74.17% of the cultured cells were immunoreactive to Bu-1a, while 58.05% and 27.37% presented IgG-IR and IgM-IR, respectively. These data indicated that the bursal primary cell cultures contained an enriched B lymphocyte population. Figure 1C–E show representative micrographs with immunoreactive cells to antibodies against Bu-1a (α-Bu-1a), anti-IgG, or anti-IgM.

### 2.2. Expression of GHRH, TRH, Ghrelin, SST, GH, and Their Receptors in BBLs

**Figure 2:**
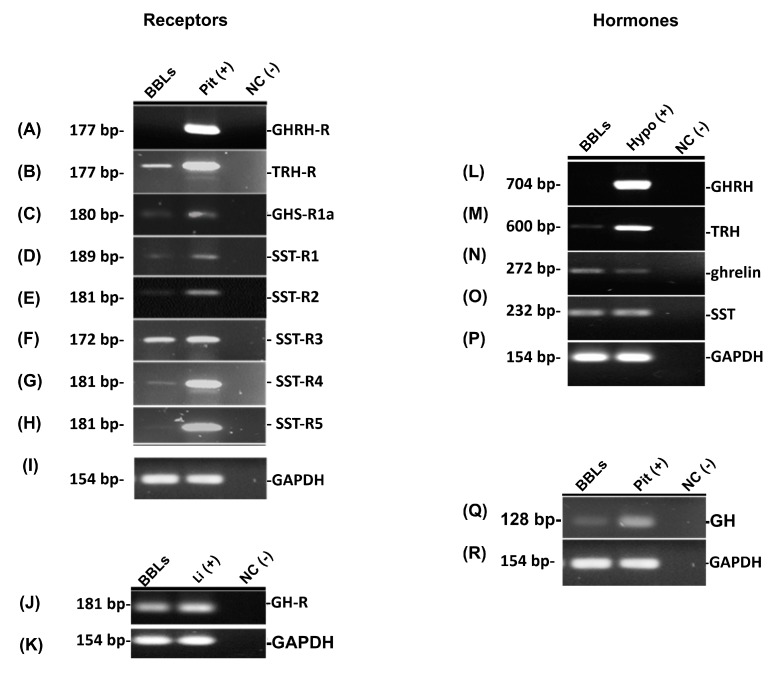
Expression of growth hormone-releasing hormone (GHRH) (**L**) and its receptor, GHRH-R (**A**), thyrotropin-releasing hormone (TRH) (**M**) and TRH-R (**B**), ghrelin (**N**) and GHS-R1a (**C**), SST (**O**) and SST-R(1-5) (**D**–**H**), GH (**Q**) and GH-R (**J**) mRNAs were evaluated in BBLs by RT-PCR and electrophoresis in agarose gels. Pituitary (Pit +), hypothalamus (Hypo +) and liver (Li +) were used as positive controls. GAPDH was used as reference gene in all cases (**I**,**K**,**R**). Base pair (bp). Negative controls (in the absence of the corresponding specific template) were included in all cases. Representative figure of 3 independent experiments.

RT-PCR determined the presence of the corresponding mRNAs for GHRH, TRH, ghrelin, SST, GH, as well as their receptors in BBLs cultures. Pituitary gland (Pit +) was used as the positive control for the expression of receptors and GH mRNAs; hypothalamus (Hypo +) as positive control for the expression of the mRNAs coding for the secretagogues; and liver (Li +) for GH-R mRNA expression. GAPDH was used as house-keeping gene in all cases. As expected, GHRH and GHRH-R mRNA expression was observed in hypothalamus and pituitary, respectively, but, interestingly, not in B-bursal cells (Figure 2A,L). In contrast, the expression of TRH and TRH-R mRNAs (Figure 2B,M), ghrelin and GHS-R1a mRNAs (Figure 2C,N), as well as SST and SST-R receptors (1–5) mRNAs (Figure 2D–H,O), were found in BBLs cultures, and also in the corresponding positive controls. Likewise, GH mRNA was expressed in BBLs and pituitary, and GH-R mRNA in BBLs and liver, respectively (Figure 2Q,J).

### 2.3. Co-Localization of GH with GHRH-R, TRH-R, GHS-R1a, and SST-R_2_ in BBLs

**Figure 3:**
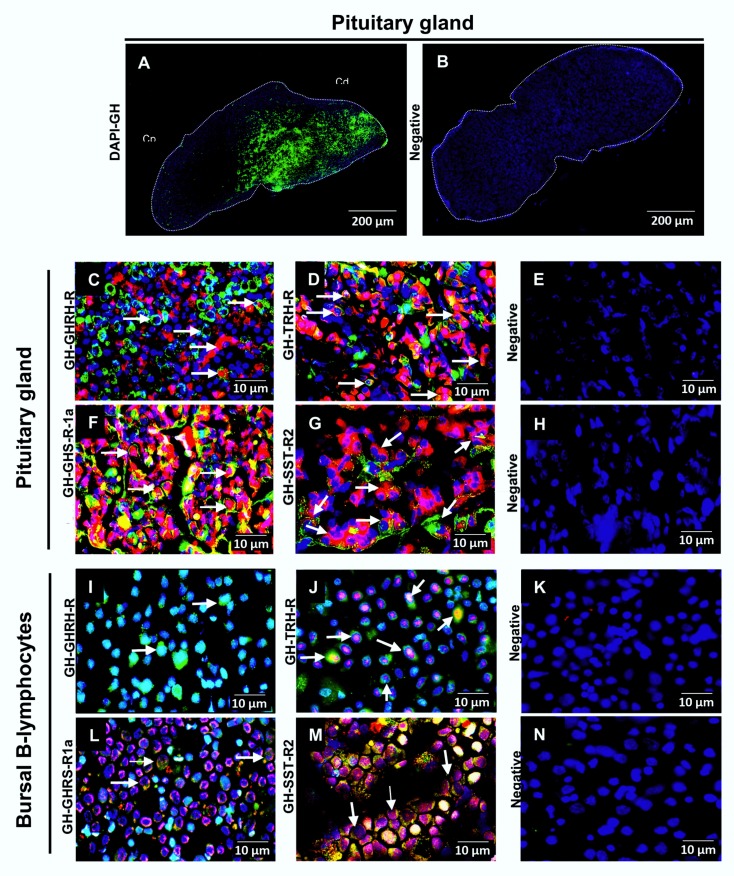
Co-localization of growth hormone (GH) and several secretagogue receptors in pituitary and BBL cultures. (**A**) As a positive control, sagittal slices of a four-week old chicken pituitary were used. Most GH-immunoreactivity (GH-IR) was located in the somatotroph cells of the caudal lobe (Cd) (green), and scarcely in the cephalic lobe (Cp). Negative controls were obtained using only Alexa 594 and Alexa 488 for both, pituitary gland and isolated BBLs (**B**,**E**,**H**,**K**,**N**). The localization of GH immunoreactivity was observed in green. On the other hand, distribution of GHRH-R, TRH-R, GHS-R1a, and SST-R_2_ immunoreactivities were observed in red; in pituitary (**C**,**D**,**F**,**G**) and BBLs (**I**,**J**,**L**,**M**). Nuclei were visualized with DAPI (blue). Arrows show immunoreactive cells to one or co-localization of both antibodies. Representative micrographs of *n* = 3 independent experiments.

Four-week-old chicken pituitaries were used as the positive control in IHC to detect GH-immunoreactivity. GH-IR cells (green) were predominantly located in the pituitary caudal lobe (Cd), as shown in Figure 3A. In the absence of primary antibody, no signal was observed in the negative control (Figure 3B).

In the chicken pituitary, GH-IR co-localized in cells that also showed GHRH-R, TRH-R, GHS-R1a, and SST-R_2_ immunoreactivities (Figure 3C,D,F,G). GH immunoreactivity was also observed in BBLs (Figure 3I,J,L,M). On the other hand, and consistent with GHRH-R mRNA absence, GHRH-R immunoreactivity was not found in BBLs (Figure 3I). However, positive immunofluorescence for TRH-R, GHS-R1a, and SST-R_2_ was clearly present in BBLs and the co-localization with GH-IR was observed for these receptors (Figure 3J,L,M). Interestingly, while GH-IR was mainly distributed in the cytoplasm, where it co-localized with GHS-R1a-IR and SST-R_2_-IR, TRH-R-IR was mainly located around the cell nuclei.

### 2.4. Effect of TRH, Ghrelin and SST Upon GH mRNA Expression in BBLs

**Figure 4:**
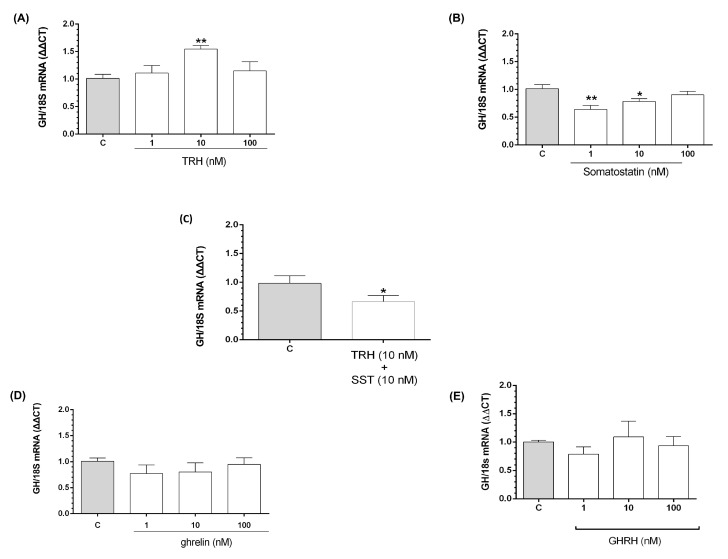
TRH, ghrelin, GHRH, and somatostatin (SST) effects upon GH mRNA expression in BBLs. After one-hour treatment with 1, 10 and 100 nM of either TRH (**A**), SST (**B**), ghrelin (**D**), GHRH (**E**), or the combination of 10 nM of both TRH and SST (**C**), the relative GH mRNA expression was determined by qPCR and corrected by the threshold cycle (CT) using the formula 2^−ΔΔCT^ [29,30]. Ribosomal 18S RNA was used as reference gene. Each bar represents mean ± SEM. Data were obtained from three independent experiments analyzed by duplicate. Asterisks indicate significant differences compared with control groups (* *p* < 0.05; ** *p* < 0.01), by using one-way ANOVA for multiple comparisons and Šidák as *post-hoc* test. A paired t test was used to evaluate the effect of TRH in combination with SST (* *p* < 0.05).

qPCR evaluated the effect of short incubation (1 h) with TRH, ghrelin, GHRH, and/or SST treatments upon GH mRNA expression in BBLs cultures. 10 nM TRH significantly increased (*p* < 0.01) GH mRNA expression by 1.54 ± 0.06-fold in comparison with the control, as shown in Figure 4A. Conversely, the treatments with 1 and 10 nM SST significantly decreased GH mRNA expression in comparison to the control group (0.64 ± 0.07 and 0.78 ± 0.04-fold; *p* < 0.01 and *p* < 0.05, respectively) (Figure 4B). In contrast, ghrelin and GHRH treatments showed no significant effects upon GH mRNA expression (Figure 4D,E). On the other hand, SST was capable of significantly inhibiting (*p* < 0.05) the TRH-induced stimulatory GH mRNA expression response (Figure 4C).

### 2.5. Effect of TRH and SST Upon GH Content and Release in BBLs Cultures

**Figure 5:**
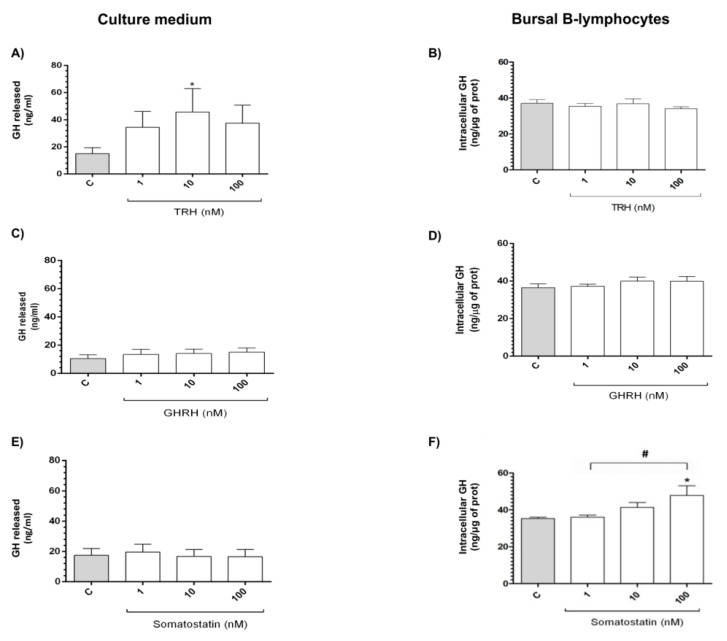
TRH, GHRH, and SST effects upon GH synthesis and release in BBLs. Intracellular (**B**,**D**,**F**) and released (**A**,**C**,**E**) GH were determined by ELISA in B-bursal lymphocyte cultures in response to TRH, GHRH, and SST (1, 10, and 100 nM). Each bar represents means ± SEM. Data was obtained from seven independent experiments for cellular fraction and five independent experiments for culture media. Asterisks represent significant differences between control group (* *p* < 0.05) using one-way ANOVA for multiple comparisons and non-parametric Dunn´s *post-hoc* test for TRH and parametric Šidák *post-hoc* test for SST. Number sign represents the differences between experimental groups (# *p* < 0.05).

ELISA quantified the intracellular GH content in BBLs and its release to the culture media after treatments with the secretagogues for 60 min. Incubations with 10 nM TRH significantly increased (*p* < 0.05) the release of GH (up to 45.69 ± 17.23 ng/mL) to the culture media (Figure 5A) in comparison to the control group (15.03 ± 4.40 ng/mL); however, no changes were observed in the intracellular GH content after TRH treatment (Figure 5B). Interestingly, GHRH had no effect on GH release or intracellular content (Figure 5C,D). On the other hand, while GH release was not changed in the culture media after SST treatment (Figure 5E) at any of the doses tested, SST (100 nM) significantly increased (*p* < 0.05, up to 47.76 ± 5.32 ng/μg) the intracellular content of GH as compared with the untreated control (35.21 ± 0.78 ng/μg of protein) and treated with 1nM (*p* < 0.05, (Figure 5F).

### 2.6. Inhibitory Effect of SST Upon TRH-Induced GH Release in BBLs

**Figure 6:**
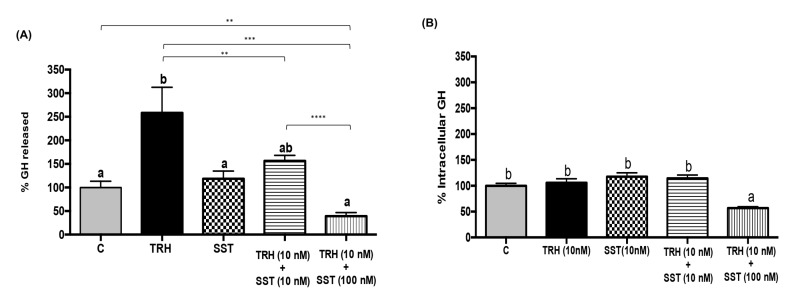
Inhibitory effect of SST upon TRH-induced GH release in BBLs. GH was determined by ELISA in either culture media (**A**) or intracellular content (**B**) in response to incubation with 10 nM TRH, 10 nM SST, or the combination of TRH (10 nM) with SST (10 or 100 nM), after one-hour treatment. Each bar represents mean ± SEM. The data were obtained from three independent experiments and expressed as percentage (%). Group with different letters are significantly different as compared with control conditions. We used one-way ANOVA with multiple comparison test and Tukey as *post-hoc* test, (*p* < 0.001). Asterisks indicate differences between experimental groups as determined by unpaired t test. ** *p* < 0.01; *** *p* < 0.001; **** *p* < 0.0001.

The effect of simultaneous administration of both TRH and SST upon intracellular GH content and release was also studied in the BBLs cultures. TRH significantly stimulated GH release (258 ± 54.2 %) in comparison to the control (100 ± 13 %), whereas SST alone had no effect (118.5 ± 16.4 %), as shown in Figure 6A. However, when co-incubated together, SST was able to prevent TRH-induced GH release in a dose-dependent fashion, reducing GH release to 156 ± 11.6 % (10 nM) and 39.1 ± 7.7 % (100 nM) when compared to the untreated control. On the other hand, incubation with TRH or SST, and the co-incubation with 10 nM TRH and 10 nM SST, had no effect upon intracellular GH content. Interestingly, when 100 nM SST was co-administered with 10 nM TRH, the intracellular GH content was significantly decreased (a reduction to 56.8 ± 2.5%, *p* < 0.001, Figure 6B) in relation to the control (100%).

### 2.7. Effect of TRH and/or SST Upon Intracellular Ca^2+^ in BBLs Cultures

**Figure 7:**
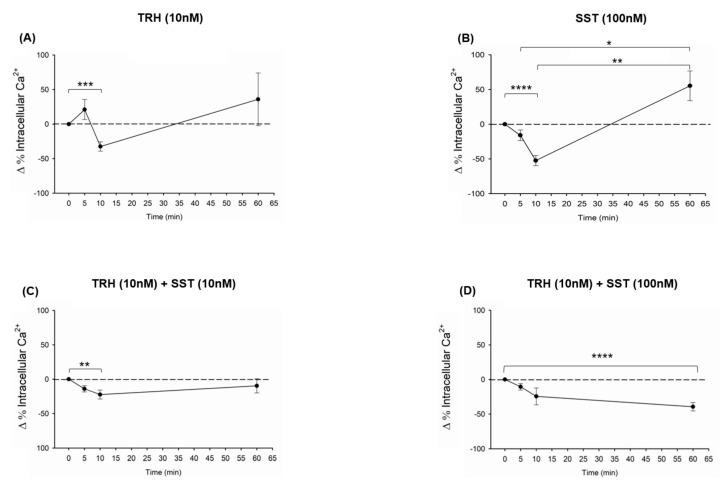
TRH and SST effects upon intracellular Ca^2+^ levels. Intracellular calcium concentrations were measured in B-bursal cells over 5, 10, and 60 min., with the following treatments: 10 nM TRH (**A**), 100 nM SST (**B**), or the combination of both hormones [TRH 10 nM + SST 10 nM (**C**) and TRH 10 nM + SST 100 nM (**D**)]. RPMI 1640 medium was used as control (0 min.). Each point represents mean ± SEM. Data was obtained from four independent experiments for the cases of TRH or SST; on the other hand, three independent experiments were used in the combination of TRH + SST. Units are expressed as a delta proportion (Δ%) in relation to the control. Asterisks indicate differences between experimental groups, as determined by paired t test. * *p* < 0.05; ** *p* < 0.01; *** *p* < 0.001; **** *p* < 0.0001.

The effects of TRH, SST, or its combination, upon changes in the intracellular Ca^2+^ concentration in BBLs cultures, were evaluated after 0, 5, 10, and 60 min. of incubation. Treatment with 10 nM TRH significantly decreased the intracellular Ca^2+^ content after 10 min. of incubation (a reduction of −32.45 ± 6.74 Δ%, *p* < 0.001 in comparison with the untreated control, whereas after 60 min. of incubation, the intracellular Ca^2+^ content showed a tendency to increase (up to 35.96 ± 38.12 Δ%) but was not significantly different with respect to the previous times (Figure 7A). Likewise, the treatment with SST (100 nM) significantly decreased the intracellular Ca^2+^ content after 10 min. incubation (a reduction of −52.37 ± 7.32 Δ %, *p* < 0.0001) as compared with 0 min. (control group) (Figure 7B); whereas after 60 min. of incubation with SST (100 nM) the intracellular Ca^2+^ content significantly increased (up to 55.3 ± 21.48 Δ %), in relation to the levels that were observed after 5 min. (−13.65 ± 6.84 Δ %, *p* < 0.029) or 10 min. (−52.37 ± 7.32 Δ %, *p* < 0.003) of treatment but not in comparison to the control group (Figure 7B). On the other hand, the combination of TRH (10 nM) + SST (10 nM) significantly (*p* < 0.006) decreased the intracellular Ca^2+^ content in BBLs after only 10 min. of incubation (a reduction of −26.32 ± 3.44 Δ %) in comparison with control (0 min.) (Figure 7C). Finally, the treatment with TRH (10 nM) + SST (100 nM) significantly (*p* < 0.0001) decreased the intracellular Ca^2+^ content in BBLs after 60 min. of incubation (a reduction of −39.51 ± 6.03 Δ %) in comparison with control (0 min.) (Figure 7D).

### 2.8. Effect of TRH and SST on CREB Phosphorylation at Ser133

**Figure 8:**
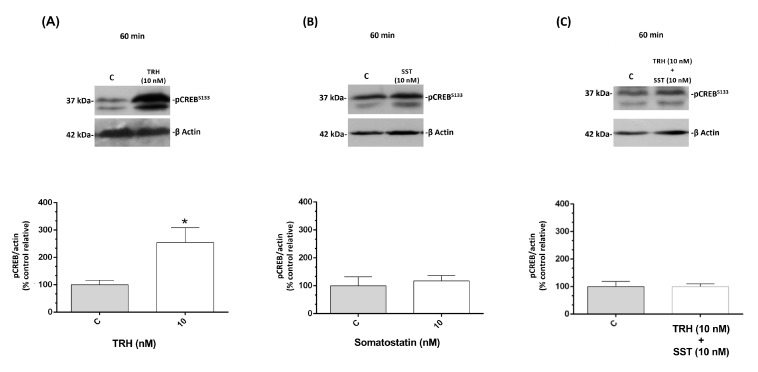
Effects of TRH and SST upon pCREB. Phosphorylation of CREB in serine-133 was analyzed after one-hour treatment with TRH (**A**), SST (**B**), or the combination of both hormones (**C**) in B-bursal cells. Representative immunoblots show the immunoreactive bands to pCREB^S133^ (37 kDa), as determined by SDS-PAGE followed by Western blot and densitometry. β Actin was used as loading and normalizing control in all cases (42 kDa). Each bar represents mean ± SEM. Data was obtained from three independent experiments and expressed as relative percentage in relation with the control group (%). The groups with asterisks are significantly different (* *p* < 0.05), as determined by paired t test.

Figure 8A shows that pCREB^S133^ significantly increased (up to 254.37 ± 53.9 %, *p* < 0.05) after 60 min. of incubation with 10 nM TRH in relation to the control group (100 ± 16.47 %). In contrast, no changes were observed in pCREB^S133^ after SST treatment (Figure 8B). Interestingly, when co-incubated together at the same doses, SST was capable of blocking the TRH-induced effect upon pCREB^S133^ and no difference was observed with the untreated control (Figure 8C).

### 2.9. Effect of TRH and SST upon BAFF mRNA Expression in BBLs Cultures

**Figure 9:**
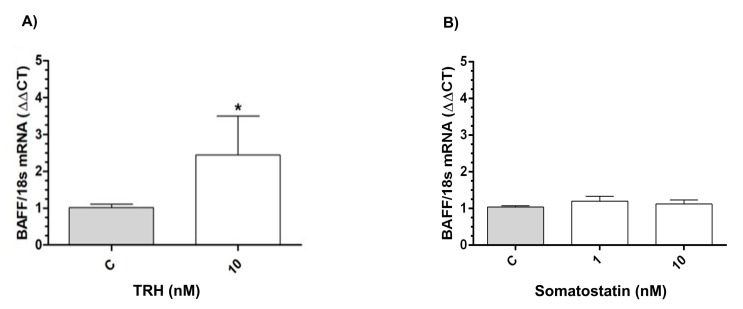
Effect of TRH and SST upon B-cell activating factor (BAFF) mRNA expression. Relative BAFF mRNA expression in BBLs after one-hour treatment with TRH (10 nM) (**A**) and SST (1 and 10 nM) (**B**) as determined by qPCR. The relative mRNA expression was corrected by the threshold cycle (CT) and using the formula 2^−ΔΔCT^ [29,30]. Ribosomal 18S RNA was used as reference gene. Each bar represents means fold ± SEM. Data were obtained from three independent experiments analyzed by duplicate. Asterisks indicate significant differences when compared with control groups (* *p* < 0.05), as determined by unpaired t test.

The incubation with 10 nM TRH induced a significant increase (1.43-fold, *p* < 0.05) in BAFF mRNA expression levels, whereas no changes were observed after incubation with either 1 nM or 10 nM SST, in comparison to the controls (Figure 9B), as shown in Figure 9A.

## 3. Discussion

The complex communication among the neuroimmune-endocrine system involves an intricate network of common chemical messengers, and their receptors, which interact through a combination of endocrine, paracrine, and/or autocrine mechanisms to exert pleiotropic effects that contribute to homeostasis [31]. Some members of the somatotropic axis, including GH, are among those commonly shared messengers between the endocrine, nervous, and immune systems. It is well known that pituitary GH plays an important endocrine role that is involved in the expression of several somatic and metabolic effects in the whole organism [32]. Additionally, it has been shown that GH is locally expressed in neural tissues, where it participates in neuroprotection and cell survival and has actions in behavior, cognition, and neurotransmission [33]. In addition, it has been described that GH and GH-R are expressed in the immune system and they exert immunomodulatory effects in several species [18,23,34], and also that GH modulates anti-apoptotic signaling pathways and survival mechanisms in B-lymphocytes [24]. Although the intimate mechanisms that regulate pituitary GH expression are rather well known, it still remains unclear how extra-pituitary GH expression is regulated. In this work, we aimed to study the potential involvement of known “classic” hypothalamic GH secretagogues in the expression and release of immune growth hormone in a primary avian lymphoid organ, the bursa of Fabricius (BF), where B-lymphocytes are produced and matured [35].

Initially, we explored whether GH and its canonical secretagogues and their respective receptors were expressed in chicken bursal B-lymphocyte cultures by RT-PCR, in comparison with hypothalamus, pituitary, and liver, respectively. As expected, the expression of GHRH, TRH, ghrelin, and SST mRNAs were found in the chicken hypothalamus used as control, confirming previous results regarding their hypothalamic production in several species, including avian models [36,37]. Additionally, the expression of GHRH-R, TRH-R, GHS-R1a, SST-Rs, and GH-R mRNAs were observed in the chicken pituitary control, in concordance with previous reports [38,39,40,41,42]; and GH-R mRNA was found in the liver. Here, we show that several components of the somatotropic axis, such as GH, TRH, ghrelin, and SST mRNAs, as well as GH-R, TRH-R, GHS-R1a, and SST-Rs (subtypes 1-5) mRNAs, are definitively expressed in chicken BBLs; however, neither GHRH nor GHRH-R mRNAs expression were detectable in these cells in our qPCR system. It has been reported in several mammalian species that GH, GHRH, TRH, ghrelin, and SST, as well as their receptors, are expressed in different immune cells, such as T- and B-lymphocytes, splenocytes, thymocytes, macrophages, and neutrophils [17,34,43].

Subsequently, we analyzed the presence and co-localization of GH with TRH-R, GHS-R1a, GHRH-R, and SST-R2 in BBLs cultures and in the chicken pituitary by immunocytochemistry. As expected, all of these receptors and GH were present in the control pituitary sections. Additionally, we found that GH-IR co-localized with TRH-R, GHRS-R1a, and SST-R2 immunoreactivities in BBL cultures. Interestingly, in these cells, TRH-R-IR was predominantly associated to the cell nuclei, whereas GH-IR was present in the cytoplasm; instead, GHRS-R1a and SST-R2 immunoreactivities were mostly located in the cytoplasm and near the membrane, where they co-localized with GH. No GHRH-R-IR was observed in BBLs, in accordance with the lack of GHRH-R mRNA expression. It has been described that these receptors are expressed in the immune system of other vertebrate groups, for example, in fish, rat, and humans, where the expression of TRH-R has been observed in thymus, bone marrow, lymph nodes, and spleen extracts [26,44,45,46]. The presence of TRH-R in other tissues besides the pituitary suggests other non-canonical actions and possibly a cross-talk between the immune and the neuroendocrine system [26].

In most vertebrates, a complex network of stimulatory (GHRH, TRH, ghrelin, PACAP [pituitary adenylate cyclase activating peptide], GnRH [gonadotropin releasing hormone]), and inhibitory (SST, IGF-1) signals regulate pituitary GH synthesis and release. Finding that the expression of several canonical GH secretagogues and their receptors co-existed simultaneously with GH in BBLs suggested that they might be involved in the regulation of local GH expression in these cells. This prompted us to study their effect, both individually and in combination, upon GH mRNA expression and GH release, and try to determine whether they had a role akin to that in the hypothalamic-pituitary axis.

It has been described that GHRH is the main stimulatory regulator of pituitary GH expression and secretion [47], and it has also been reported to stimulate GH expression in chicken testis [48] and in quail immortalized neuroretinal (QNR/D) cells [49]. The expression of GHRH-R was previously reported in thymocytes and splenocytes of rats [50,51], fish [52], and in human T-lymphocytes [53]. Interestingly, neither GHRH nor GHRH receptor mRNAs, nor GHRH-R protein, were detected in chicken BBLs, in accordance with previous findings, where the absence of GHRH and GHRH-R in the chicken spleen was reported [41,54]. Additionally, the incubation of BBLs with GHRH (1–100 nM) had no effect upon either GH release or GH mRNA expression. These results open an intriguing question regarding the reasons why GHRH-R is not expressed in the immune system of the chicken, which deserves to be further explored.

Ghrelin and its canonical receptor GHS-R1a have a ubiquitous distribution that includes gastroenteric, nervous, reproductive, and immune systems [55]. Ghrelin and GHS-R expression were both reported in human B-cells, T-cells, and neutrophils, as well as in several human leukemic cell lines [56]. GHS-R1a expression has been previously reported in chicken immune tissues, such as thymus [57], spleen [40,57], and in the BF [58]. In this work, we found co-expression and co-localization of GHS-R1a and GH in BBLs, suggesting the participation of ghrelin and GHS-R1a upon immune GH regulation. However, we did not observe any effect of ghrelin upon GH mRNA expression at the doses tested, or upon the release of GH to the culture media (data not shown). Thus, it is possible that ghrelin/GHS-R exerts other GH-independent immunomodulatory effects [56] in these cells.

In chickens, TRH is a potent and effective GH secretagogue that affects the release of this hormone from pituitary cells [59,60]. Here, we showed that TRH (10 nM) was able to significantly increase both the expression of GH mRNA and GH release to the culture media in BBLs, suggesting that it could act as a local GH secretagogue in these cells. These results were analogous to those that were reported in neuroretinal cells, where TRH stimulated GH release to the culture media and significantly reduced the intracellular GH content after short-term (15 min.) experiments, while the content of GH increased in the cell as well as in the media after long-term (48 h) incubation, although at a higher dose (1 µM) than in the present study [47]. In contrast, the intracellular content of GH in BBLs was not significantly different under the conditions that were employed here.

Hypothalamic somatostatin is well recognized as the main inhibitory regulator of both basal and stimulated pituitary GH secretion in vertebrates [61,62]. Furthermore, SST is known to be widely distributed in cells from the endocrine, neuroendocrine, neural, gastrointestinal, vascular, and immune systems, where it exerts inhibitory action on numerous physiological functions, including the secretion of several hormones, neuropeptides, and cytokines, among others [61]. In chicken pituitary cells, it is well established that SST inhibits GH release through the SST-R2 [63]. In this work, we corroborated the mRNA expression of SST and its five receptor subtypes (SST-R1 to SST-R5) in BBLs, and specifically co-localized the presence of SST-R2 and GH in both chicken pituitary and BBLs. Likewise, the expression of SST-Rs has also been reported in other chicken tissues, including the spleen [34]. We found that, similar to what happens in chicken somatotrophs when co-incubated with TRH [64], SST significantly decreased TRH-induced GH release in a dose-dependent manner, and considerably diminished the intracellular content of GH in BBL cultures, although only at the highest dose tested (100 nM). However, when administered alone, SST had no effect on basal GH release at any dose, similar to the results that were observed in human B-lymphocytes (in vitro), where SST and its analogue “SMS 201-995” did not have any effect on the release of GH to the culture media [65,66]. Additionally, by itself, SST significantly increased basal intracellular GH content in BBLs. Interestingly, our experiments demonstrated that, when administered alone at low doses, SST was able to significantly inhibit basal GH mRNA expression in BBL cultures. Furthermore, when co-incubated together, SST also considerably blocked TRH-induced GH mRNA expression in these cells, which probably explains the reduction of intracellular GH content that is mentioned above. These results indicate that, in these immune cells, SST might be directly involved in inhibiting GH mRNA transcription, and not only GH release as has been traditionally described in somatotrophs. There are reports where SST (or its analog, octeotride) has been shown to reduce GH mRNA expression, and this might be mediated by SST-R2 in GH-secreting GC cells [67]; by reducing the steady state levels of GHRH [68] in rats; or, by interfering with GHRH-induced GH gene transcription at the level of adenylate cyclase through inhibitory G protein in MtT/S cell line [69]. The mechanisms that are involved in SST-induced reduction of GH mRNA expression in B-lymphocytes deserve further research.

The inhibitory role of somatostatin upon secretagogue actions has been extensively documented in pituitary cells [70]. In the pituitary, TRH binds to TRH-R (Gq protein associated receptor) and then activates phospholipase C (PLC) and protein kinase C (PKC); in turn, PKC phosphorylates transcription factors, such as CREB [71]. The phosphorylation of CREB (in Ser 133) strongly promotes GH transcription [4]. On the other hand, SST can promote the inhibition of PKC and PKA [72].

In immune cells, GH expression due to CREB activation remains unclear. In this work, we observed that TRH significantly increased pCREB^S133^ as well as GH mRNA expression. Conversely, SST decreased both basal and TRH-induced GH mRNA expression, and blocked TRH-stimulated phosphorylation of CREB, but no changes were observed upon pCREB^S133^ when applied alone. These results suggest that GH mRNA expression in BBLs could be mediated by TRH-induced pCREB^S133^ and that SST inhibits this stimulatory effect by interfering with CREB phosphorylation. However, it is possible that other transcription factors involved in the SST signaling pathway could be activated in order to decrease GH expression since SST by itself also decreased GH mRNA expression without changes in pCREB^S133^. In other studies, it has been shown that SST inhibits cAMP production that is induced by corticotropin-releasing hormone (CRH) and GHRH in primary pituitary cell cultures [73]. Furthermore, SST inhibits the effect of forskolin on cAMP production, PKA activation, CREB phosphorylation, and transcription [74].

Intracellular Ca^2+^ is a second messenger involved in multiple processes such as cell metabolism, endo- and exocytosis, vesicular transport, neurotransmission, growth, and homeostasis [75]. The complex and fine-tuned Ca^2+^ dynamics occur in variable timeframes (from milliseconds to minutes), in which effector or/and inhibitory molecules are activated, blocked, and biotransformed to control a wide variety of physiological functions. In the pituitary, Ca^2+^ has a pivotal role in hormonal synthesis and secretion, in which hypothalamic hormones, such as GHRH, GnRH, TRH, CRH, and SST, are involved in an intricate system that includes transport and well-orchestrated changes between different cellular compartments [38]. Our study shows, for the first time, that TRH and SST exert actions upon intracellular Ca^2+^ levels in BBLs. We found that BBLs that are treated with either TRH or SST showed a specific response pattern that includes an initial decrease of Ca^2+^ concentration at 5–10 min. after treatment, followed by an increase at 60 min. post-treatment for both peptides. Interestingly, this late Ca^2+^ upregulation that occurs after the initial decrease was completely abolished by TRH + SST co-treatment, which positively correlates with the simultaneous inhibitory effect that was observed upon GH mRNA expression and secretion induced by TRH + SST at 60 min. after treatment. The associated changes of intracellular Ca^2+^ distribution/concentration and its correlation with endocrine GH production and secretion have been characterized only in shorter times [76,77,78,79] in comparison with our experimental protocol despite the evidence about TRH and SST as potent GH synthesis and release regulators in the pituitary. Additionally, in human B-lymphoblast cells, SST treatment resulted in a fast and short Ca^2+^ increase at 20 s. [80]. These results indicate that Ca^2+^ is involved during the inhibitory effect of SST and the stimulatory effect of TRH upon GH mRNA expression and protein release in BBLs, but understanding of the intimate mechanisms involved requires further investigation

BAFF, which is an important immune cytokine, activates humoral responses in chickens infested with the infectious bursal diseases virus (IBDV) [81] and it is involved in the negative selection of B-lymphocytes [82]. We found a stimulatory effect of TRH on BAFF mRNA expression in BBLs, suggesting an immunomodulatory function for the TRH-GH mini-axis. In that way, TRH could promote BAFF mRNA expression to modulate immune and inflammatory responses, since it has been reported that TRH can promote (in vivo and in vitro) interleukin production, such as IL-1, IL-6, IL-10, and TNFα or INFα/γ [26]. Local actions for TRH and GH in immune cells suggest a complex network of cellular and molecular interactions that represent an emerging field for research.

In summary, the presence of TRH and SST and their corresponding receptors in BBLs suggest their involvement in the regulation of local GH expression and a role in immunomodulatory responses. According to our results, TRH and SST are involved as regulatory factors in the expression and release (in vitro) of GH, in pCREB^S133^ involving specific and time related intracellular Ca^2+^ changes. As TRH increased GH and BAFF mRNA expression, it is likely to have immunomodulatory actions. This work provides evidence about the existence of a complex interaction between somatotropic regulatory elements in the immune system that involves autocrine, paracrine, and endocrine mechanisms, which may have important participation in the modulation of the immune response in vertebrates. It also contributes to shedding light regarding the intrincate cross-talk between common peptide messengers of the neuroimmune-endocrine system.

## 4. Material and Methods

### 4.1. Animals and Tissues

Pilgrim’s México donated all chickens used in this study. Broiler chickens were kept on a 12L:12D photoperiod with ad libitum access to commercial food (Caporina^®^Initiator-Api-Aba [1–14 days of age]; Caporina^®^Growth-Api-Aba-Premium [15–28 days of age], MaltaCleyton-ADM, Mexico), and water in the vivarium at the Institute of Neurobiology of the National Autonomous University of México (UNAM). The birds were killed by decapitation following the protocol that the Institute’s Bioethics Committee approved (number 038/19 October 2010), and the organs used in this study (bursa of Fabricius [BF], pituitary, brain, and liver) were collected from four-week old chickens. The hypothalamus was dissected out from the brain while using as reference marks the third ventricle, the optic chiasm (rostrally), and the mammillary bodies (caudally).

### 4.2. Hormones and Antibodies

The following peptides were employed in the preparation of treatments: TRH (pGlu-His-Pro-Amide, P-2161-SIGMA, St. Louis, MO, USA), ghrelin-acylated (Human-G-3902-SIGMA), SST (SRIF-S-9129-SIGMA), and growth hormone-releasing factor (G-8895-Lot41K49501-SIGMA). The recombinant chicken growth hormone (rcGH) was used in ELISA standard curve (American Cynamid, Princenton, NJ, USA). Table 1 describes the primary and secondary antibodies used in this study.

### 4.3. Primary B- Bursal Lymphocytes (BBLs) Cultures

BFs were aseptically removed, as described elsewhere [22,24]. Immediately, the BFs were minced in RPMI 1640 medium (ThermoFisher Scientific, Carlsbad, CA, USA) and dispersed while using a glass homogenizer. Cell suspension was filtered through 100 µM nylon mesh (twice) and then centrifuged at 1800 rpm for 5 min. The cell suspensions were resuspended in RPMI 1640, and cell number and viability were determined while using a hematocytometer and trypan blue [83]. Each BBL culture was obtained from at least 10 BFs. Cells (4 × 10^6^ BBLs) were placed in Eppendorf tubes as cell suspension in 500 µL of RPMI 1640 at 37 °C in a humidified chamber with 95% air and 5% CO_2_ atmosphere, and then incubated for 60 min. 

### 4.4. Treatments

For treatments, the secretagogues GHRH, TRH, SST, and ghrelin-acylated were diluted in RPMI 1640. BBL cultures were treated with either RPMI 1640 medium as control; ghrelin; TRH; SST; or, a combination of TRH + SST. For all of the treatments, we performed dose-response curves while using 1, 10, and 100 nM of each peptide, while, for combinations, the following were used: 10 nM TRH + 10 nM SST or 10 nM TRH + 100 nM SST. The BBL cultures were incubated with the corresponding treatments for 60 min. at 37 °C in a humidified chamber with 5% CO_2_. After incubation with the treatments, BBLs were separated from culture media by centrifugation (1800 rpm for 5 min. at 4 °C). The intracellular calcium concentration changes were determined after 0, 5, 10, and 60 min. of incubation, as described below.

### 4.5. Immunohistochemistry and Immunocytochemistry

BFs and pituitaries (as GH-positive controls) were fixed with the Bouin–Hollande sublimate [22] for 24 h, dehydrated in ethanol, and then embedded in paraffin wax. Tissue sections of 4–8 μm were then cut while using a rotatory microtome (Leica, RM2135 model, D-35578 Wetzlar, Germany) and mounted onto charged glass slides (Superfrost/Plus, Fisher, Pittsburgh, PA, USA). On the other hand, BBLs (3 × 10^5^) were deposited on charged slides by cytospin (Thermo Scientific 4 Cytocentrifuge, Millersburg, OH, USA) centrifugation (1000 rpm) and then fixed with 4% paraformaldehyde (PFA) for 30 min., and then the slides were washed and stored in Tris-buffered saline (TBS) buffer.

Serial tissue sections of BFs and pituitaries were cleared in xylene (Fisher Scientific, Millersburg, OH, USA) 3 × 5 min., rehydrated in a graded series of ethanol (absolute alcohol, 95%, 70% and 50% alcohol), then soaked for 2 min. in lugol, followed by 4 min. in thiosulphate and finally rinsed in distilled water [22]. Tissues and BBLs slides were washed in TBS (3 × 10 min.) and permeated with citrate buffer at 80 °C for 30 min., then free binding sites were blocked with 5% non-fat dry milk (Bio-Rad, Hercules, CA, USA) for 1 h. After blocking, the slides were washed with TTBS (0.1% Triton X-100 in TBS) 3 times, and the tissues and BBLs slides were incubated overnight with the respective primary antibodies (Table 1). All of the slides were washed (3 × 10 min.) and incubated with the secondary antibodies (Table 1) and DAPI (4′,6-diamidino-2-phenylindole) to label cell nuclei. All of the slides were mounted with vectashield (Vector Laboratories Inc., Burlingame, CA, USA) and images were captured with a Zeiss LSM 780 DUO (Carl Zeiss AG, Oberkochen, Germany) confocal microscope. Image processing was performed while using ImageJ software (developed by NIH, freeware).

### 4.6. RNA Isolation and cDNA Synthesis

The total RNA was extracted from BBLs, pituitaries, hypothalamus, and liver with Direct-zol RNA MiniPrep Plus (Zymo Research, Irvine, CA, USA) kit, according to the manufacturer’s instructions. cDNA was synthetized by reverse-transcription while using 1 µg of total RNA in a final volume of 40 µL. Reverse transcriptase (M-MLV Reverse transcriptase, Promega, 200 U/µL) reaction was performed following previous reports [84].

### 4.7. RT-PCR

Amplification was performed from 1 µg of cDNA and thermocycling conditions as follows: 35 cycles (denaturation: 95 °C for 30 s; annealing: 58 °C for 30 s; extension: 72 °C for 1 min.; and, final extension step: 72 °C for 7 min.) in a thermocycler (2400 Perkin-Elmer thermocycler, Foster City, CA, USA). We used the following mix to a final volume of 40 µL containing: 5*X* PCR buffer, 2 mM MgCl_2_, 10 mM dNTPs mix, 5 U/µL Taq DNA polymerase, and 0.1 mM specific oligonucleotide primers (Table 2). Glyceraldehyde-3-phosphate dehydrogenase (GAPDH) was used as a reference gene. The negative controls were carried without cDNA templates. PCR products were resolved by constant voltage electrophoresis at 100 V in 1% (*w*/*v*) agarose gels with 1*X* TAE buffer while using Bio-Rad electrophoresis chambers (Hercules, CA, USA) and they were visualized by ethidium bromide staining.

### 4.8. Quantitative PCR (qPCR)

GH mRNA expression was measured by real time PCR (qPCR) in a StepOne Thermocycler Real-Time PCR system (Applied Biosystems, Foster, CA, USA), while using Maxima SYBR Green qPCR Master Mix (2*X*) (ThermoFisher Scientific, Waltham, MA, USA) in a final volume of 10 µL containing: 3 µL cDNA (1:10 dilution) and 0.5 µM of each specific primer (Table 2). The reactions were performed under the following conditions: initial denaturation at 95 °C for 10 min., followed by 45 cycles of 95 °C for 15 s, 60 °C for 30 s, and 75 °C for 30 s. The relative content of GH and BAFF mRNAs were calculated with the comparative threshold cycle (Ct) method and while using the formula 2^−ΔΔCT^ [29], where gene expressions were relative to the geometric mean of 18s mRNA [30].

### 4.9. SDS-PAGE/Western Blot of pCREB^S133^

BBLs (4 × 10^6^) were homogenized by sonication (Cole-Parmer 130-Watt Ultrasonic Processors 44347, GE-130PB, Vernon Hills, IL, USA) in a protease inhibitor cocktail (Mini-complete, Roche, Basel, Switzerland) that was diluted in 0.05 M HCl-Tris, pH 9.0. The total proteins were determined by the Bradford micro-method (Bio-Rad, Hercules, CA, USA).

The samples (containing 80 µg protein) were analyzed by sodium dodecyl sulfate-polyacrylamide gel electrophoresis (SDS-PAGE) in 1.0 mm thick, 6 cm long, 12.5% gels, while using the buffer system of Laemmli, 1970 in a mini-Protean II cell (Bio-Rad, Hercules, CA, USA) [85]. Samples were electrophoresed under reducing conditions (in presence of 5% (*w*/*v*) 2-mercaptoethanol). After electrophoresis, the gels were equilibrated in transfer buffer (25 mM Tris–HCl, 192 mM glycine, 20% (*v*/*v*) methanol, pH 8.3, for 30–60 min.), and then electrotransferred (at 200 mA for 60 min.) to nitrocellulose membranes (Bio-Rad). Later, the membranes were washed with TBS (30 mM Tris, 500 mM NaCl pH 7.5) and then blocked with 5% (*w*/*v*) non-fat dried milk (Bio-Rad) in TBS for 1 h at room temperature. After blocking, the membranes were washed with TTBS (TBS containing 0.05% (*v*/*v*) Tween 20) and incubated overnight at 4 °C temperature with anti-pCREB antibody (1:5000 dilution). Subsequently, the membranes were rinsed in TTBS and incubated for 2 h with the secondary antibody (goat anti-rabbit-HRP), and then diluted 1:5000 in TTBS. pCREB-immunoreactive (IR) bands were developed by incubating the membranes in ECL chemiluminescent reagent (Amersham-Pharmacia, Buckinghamshire, UK) for 30 min., and then exposed to Kodak Biomax ML film. Luminograms were analyzed by densitometry while using Image Lab Software (Bio-Rad, Hercules, CA, USA). The stripping method that was described by Negritto and Manthey, 2016 [86] was used when pCREB immunoreactivity was normalized with β-actin-IR.

### 4.10. GH ELISA

GH immunoreactivity was quantified in BBLs protein extracts (80 µg) and in 25 µL of culture media concentrate (final volume of 50 µL obtained from 1 mL dialyzed and concentrated culture media in Amicon Ultra 4 mL Centrifugal Filters [Merck Millipore Ltd.Tullagreen, Carringtwohill, Co.Cork, IRL] in an Avanti J-25 centrifuge [Beckman Coulter, Inc. Palo Alto, CA.]), while using an indirect enzyme-linked immunosorbent assay (ELISA) [87,88]. Briefly, 96-well microtiter plates (Immulon 2HB, Flat Bottom Microtiter Plates by Thermo Scientific) were coated overnight with 12 ng rcGH/per well in 100 µL carbonate buffer (1 M), pH 10.3, at 4 °C. After antigen coating, the plates were washed with TPBS (0.01 M sodium phosphate, 0.15 mM NaCl, 0.05% *v*/*v* Tween 20, pH 7) while using a manual microplate immune-washer (ENE, lowboy, Nalgene; Nunc-Immuno Wash 12, InterMed Nunc). This procedure was performed after each incubation step. The samples and serial dilutions of rcGH (0.5–1250 ng/mL) diluted in TPBS were then incubated for 16 h with 100 µL of primary antibody (α-cGH, AFP-551-11-1-86-National Hormone and Pituitary Program, Torrance, CA) diluted 1:100,000 with TPBS and 1% *w*/*v* nonfat dried milk. The samples and standards (100 µL) were then added to coated wells and incubated for 2 h at room temperature. After incubation, secondary antibody (horse-radish peroxidase-anti-rabbit IgG conjugate) diluted 1:5000 with TPBS and 1% *w*/*v* nonfat dried milk was added and incubated for 2 h at room temperature. The colorimetric reaction was generated while using 2,2-amino-di-(3-ethylbenzothiazoline sulfonate) as substrate (Sigma, Saint Louis, MO, USA), and the plates were read in an ELISA microplate Reader (Bio-Rad), at a wavelength of 405 nm.

### 4.11. Quantification of Intracellular Ca^2+^

BBLs (4 × 10^6^) were collected at 0, 5, 10, and 60 min. after treatments and homogenized by sonication (Cole-Parmer 130-Watt Ultrasonic Processors 44347, GE-130PB, Vernon Hills, IL, USA) in 100 µL of lysis buffer (100 mM Tris, pH 7.5) in order to quantify the intracellular Ca^2+^ concentration. The homogenates were centrifuged at 10,000× *g* for 15 min. at 4 °C (Sorvall Legend micro 21R Centrifuge, Thermo Scientific). The supernatant was collected and stored on ice; all of the samples were analyzed in the same day while using a Ca^2+^ Colorimetric Assay Kit (Sigma, MAK022, St. Louis, MO, USA and Abcam, ab102505, Cambridge, UK), following the manufacturer’s instructions.

### 4.12. Statistical Analysis

In all of the experiments, the values are expressed as mean ± standard error (SEM). Significant differences between groups or treatments were determined by either Student’s t test or one-way ANOVA analysis, followed by parametric (Šidák, Dunnet, Tukey) or non-parametric (Dunns) *post-hoc* tests. *p*-values less than 0.05 were determined to be statistically different (* *p* < 0.05; ** *p* < 0.01; *** *p* < 0.001; **** *p* < 0.001).

## 5. Conclusions

This work shows that GH, TRH, ghrelin, SST and their corresponding receptors are expressed in bursal B-lymphocytes. TRH increased GH mRNA expression and GH release, and also stimulated BAFF mRNA expression and phosphorylation of CREB. On the other hand, SST decreased GH mRNA expression and increased intracellular GH content. In addition, SST significantly prevented TRH-induced GH mRNA expression and GH release in BBL cultures. Interestingly, GHRH and GHRH-R were not expressed in bursal B-lymphocytes, and GHRH did not stimulate GH mRNA expression or GH release in these cultures. Both TRH and SST provoked changes in intracellular Ca^2+^ levels. Our results suggest that TRH and SST are implicated in the regulation of GH expression and release in BBL cultures; and it is likely that these peptides exert autocrine/paracrine immunomodulatory actions involved in the maturation of B-lymphocytes.

## Figures and Tables

**Table 1:** Antibodies.

Target	Host/Type	Dilution	Source	Cat. No.
cGH	rabbit/polyclonal	1:100,000	NHPP	AFP-551-11-1-86
cGH	guinea pig/polyclonal	1:2000	Washington Biotech.	GP-SHA-1
GHRH-R	rabbit/polyclonal	1:300	Abcam	ab28692
TRH-R	rabbit/polyclonal	1:300	Abcam	ab72179
SST-R2	rabbit/polyclonal	1:300	Sta Cruz Biotechnology	sc-25676
GHS-R1a	rabbit/monoclonal	1:300	Sta Cruz Biotechnology	sc-20748
p^s133^-CREB	Rabbit monoclonal	1:5000	Abcam	ab32096
chicken/Turkey IgG	rabbit/polyclonal	1:100	Zymed	GI-3100
chicken Bu-1a-FITC	mouse/monoclonal	1:500	Southern Biotech	8365-02
chicken-IgM Antibody-FITC	goat/polyclonal	1:500	Rockland	603-102-007
β-actin	mouse/monoclonal	1:1000	Sta Cruz Biotechnology	SC-47778
Alexa Fluor 594 anti-Rabbit-IgG	goat/polyclonal	1:2000	Invitrogen	A11012
Alexa 488 anti-Rabbit-IgG	goat/polyclonal	1:2000	Invitrogen	A11078
Alexa 488 anti-guinea pig IgG	goat/polyclonal	1:2000	Invitrogen	A-11073
Goat anti-Rabbit IgG (H+L) Cross-Adsorbed Secondary Antibody	goat/polyclonal	1:5000	ThermoFisher scientific	G-21234
Goat anti-Mouse IgG (H+L) Cross-Adsorbed Secondary Antibody, HRP	goat/polyclonal	1:5000	ThermoFisher scientific	G-21040

**Table 2:** Oligonucleotides.

Target	Primer	Sequence (5′-3′)	Product Size	Accession Number
**Ligands**				
cGHRH	Fwd	TAC CTG AGT GGG AGC TGA TC	704	>NM_001040464.1
	Rev	CAT CAG TCT CCA GCT GGT CA		
cTRH	Fwd	ATT AAA CAT GCC TCT GCC ACA	600	>XM_025154454.1
	Rev	AAA CAA TTA CTT TCT CAT TCC TCT G		
cGhrelin	Fwd	CATACAGCAACAAAAGGATAC	272	>NM_001001131.1
	Rev	TGTGGTTGTCCTTCAGCT		
cSST	Fwd	CACCTGTCCTCCCCATCCAC	232	>NM_205336.1
	Rev	CGGAGTGCATGTCACGCAAG		
cGH	Fwd	CGCACCTATATTCCGGAGGAC	128	>NM_204359.2
	Rev	GGCAGCTCCATGTCTGACT		
cBAFF	Fwd	GATCTCAGCTTGGTGACATTAT	140	NM_204327.2
	Rev	TTAGCTCTTCTTCGTGGTATTG		
**Receptors**				
cGHRH-R	Fwd	GCTGGTCAGAGCCATTCCCTT	177	>NM_001037834.2
	Rev	AGCGTAGCCTCCTGAATGCCA		
cTRH-R	Fwd	ATGCCCTATCGAACACTGGT	177	>NM_204930.1
	Rev	ATGGCAGTTGCAGAGTTTCCT		
cGHS-R	Fwd	TGGCCTTCTCCGACCTGCT	180	>NM_204394.1
	Rev	TGGCGACGTACCGCTCCAC		
cSSTR-1	Fwd	CCGTGGCTAAGATGGTCAACCT	189	>NM_001113167.1
	Rev	GCAGCAAGAAGCCCATCAGGA		
cSSTR-2	Fwd	GGCACCGGTATGTAGGGAGTC	191	>XM_015279869.2
	Rev	ATGCGTGCTGCCACATGGG		
cSSTR-3	Fwd	GGCACCGGTATGTAGGGAGTC	172	>XM_015286025.2
	Rev	ATGCGTGCTGCCACATGGG		
cSSTR-4	Fwd	GGCCATGTTCGTTGTCTGCT	181	>XM_015283378.2
	Rev	GCCGTGGAAAGAGTGCCGGA		
cSSTR-5	Fwd	CCGCTACCTGGCAGTAGTTCA	156	>XM_015294246.2
	Rev	TTGCAGGTGTGAAAGTCCTCC		
cGH-R	Fwd	ACTTCACCATGGACAATGCCTA	181	>NM_001001293.1
	Rev	GGGGTTTCTGCCATTGAAGCTC		
**Reference genes**				
cGAPDH	Fwd	TGTGGAGAGATGGCAGAG	154	>NM_204305.1
	Rev	GTCAGGTCAACAACAGAGAC		
c18S	Fwd	CTCTTTCTCGATTCCGTGGGT	100	>XR_003078044.1
	Rev	TTAGCATGCCAGAGTCTCGT		
